# Association between caffeine intake from foods and beverages in the diet and hearing loss in United States adults

**DOI:** 10.3389/fneur.2024.1436238

**Published:** 2024-07-24

**Authors:** Fei Xia, Yuanyuan Ren

**Affiliations:** Department of Otorhinolaryngology-Head & Neck Surgery, Beijing Chaoyang Hospital, Capital Medical University, Beijing, China

**Keywords:** hearing loss, dietary, caffeine intake, speech-frequency hearing loss, high-frequency hearing loss, NHANES

## Abstract

**Background:**

Hearing loss (HL) is the third most prevalent condition, significantly affecting individuals and society. Recent research has explored the potential impact of nutrition, particularly caffeine intake, on HL. While some studies focus on coffee, caffeine intake should be assessed across all dietary sources. This study examines the association between dietary caffeine intake and HL.

**Methods:**

Our cross-sectional study included 6,082 participants from the National Health and Nutrition Examination Survey (NHANES). Participants were divided into two groups based on their median caffeine intake: low and high. The study investigated two types of HL: speech-frequency hearing loss (SFHL) and high-frequency hearing loss (HFHL). Binary logistic regression analyzed the correlation between caffeine intake and HL, and a restricted cubic spline (RCS) model assessed potential non-linear associations. Subgroup analyses were also conducted.

**Results:**

High caffeine intake was associated with significantly higher rates of SFHL and HFHL compared to low intake (SFHL: 15.4% vs. 10%, HFHL: 30.5% vs. 20.6%, both *p* < 0.001). Unadjusted logistic regression showed a higher likelihood of SFHL (OR[95%CI] = 1.65[1.41–1.92]) and HFHL (OR[95%CI] = 1.69[1.50–1.90]) in high caffeine consumers. After adjusting for confounders, high caffeine intake remained significantly associated with SFHL (OR[95%CI] = 1.35[1.09–1.66]) but not HFHL (OR[95%CI] = 1.14[0.96–1.35]). The RCS model indicated a linear increase in the risk of SFHL and HFHL with higher caffeine intake (non-linear *p* = 0.229 for SFHL, *p* = 0.894 for HFHL). Subgroup analysis revealed that increased caffeine intake was linked to higher SFHL and HFHL risks in participants under 65 years but not in those 65 years and older (SFHL: *p* for interaction = 0.002; HFHL: *p* for interaction <0.001).

**Conclusion:**

Our study indicates a strong correlation between dietary caffeine intake and the risk of HL in American adults, particularly those under 65. High caffeine intake was linked to an increased risk of SFHL, but not HFHL, after adjusting for relevant variables.

## 1 Introduction

Hearing loss (HL) is a common ailment that can have a significant impact on many aspects of a person’s life if it is not treated or supported in terms of communication needs (1). As per the 2019 Global Burden of Disease (GBD) research conducted by the World Health Organization, HL ranks third globally in terms of causing disability, impacting 157 million individuals. According to projections, 2.45 billion people will have some kind of HL by 2050, and at least 700 million of them would need rehabilitation programs (1, 2).

Pathological hearing loss can result from lesions in the external auditory canal, middle ear, cochlea (which includes hair cells, spiral ganglion cells, and the stria vascularis), and auditory nerve (2–4). Recent research has indicated that systemic inflammation, altered microcirculation, and free radical production may contribute to cochlear injury and subsequent HL (5–8). Moreover, a wide range of factors affect the incidence and severity of HL. The risk and progression of HL have been linked to a number of factors, including exposure to loud noise, socioeconomic difficulties, medical conditions such as the use of ototoxic medications, ear infections, hypertension, and diabetes, genetic predispositions, and hormonal influences, particularly estrogen levels (7, 9–11).

Traditional risk factors help us understand the causes of HL, but they do not fully take into account the complexity of the condition (12). Recently, an increasing number of studies have shown that diet is associated with HL, which can be attributed to specific dietary patterns or specific components of the diet (13–17). And research has demonstrated that diet exerts a dual effect on the risk of HL, with certain dietary components shown to reduce the risk of HL (18–28), while others have been proven to increase the likelihood of developing HL. A higher consumption of n-3 fatty acids (18, 19), oily fish (18–20), magnesium (21), and various vitamins (21–24), as well as antioxidants like β-carotene (21) and moderate alcohol use (25), has been linked to a reduced risk of hearing loss. On the other hand, a diet rich in cholesterol (26), foods with a high glycaemic load (27), and excessive alcohol intake (28) have been shown to be connected with a higher risk of HL.

The most widely used psychostimulant is caffeine (29), which is found naturally in coffee beans, tea leaves, cocoa beans, and kola nuts. It is also added to a variety of meals and drinks. The main food sources are tea, coffee, yerba mate, and caffeinated sodas (especially cola varieties) and energy beverages (30). Caffeine acts as a stimulant to the central nervous system; its biological effects indicate that it affects the peripheral and central auditory systems, including the inner ear directly (31). Despite these links, it’s still unclear how exactly caffeine affects hearing and whether it’s positive or negative (32–34).

There has not been much research done on the relationship between dietary caffeine intake and HL in the general population up until now. Although there is some research on coffee intake and HL, caffeine intake does not depend on coffee alone, but should be considered in the context of caffeine intake from all foods and beverages in the diet (35). In order to close that gap, this study uses information from the National Health and Nutrition Examination Survey (NHANES) to investigate the association between adult population in the United States’ caffeine intake from foods and beverages in the diet and HL.

## 2 Methods

### 2.1 Study design and population

**Figure 1:**
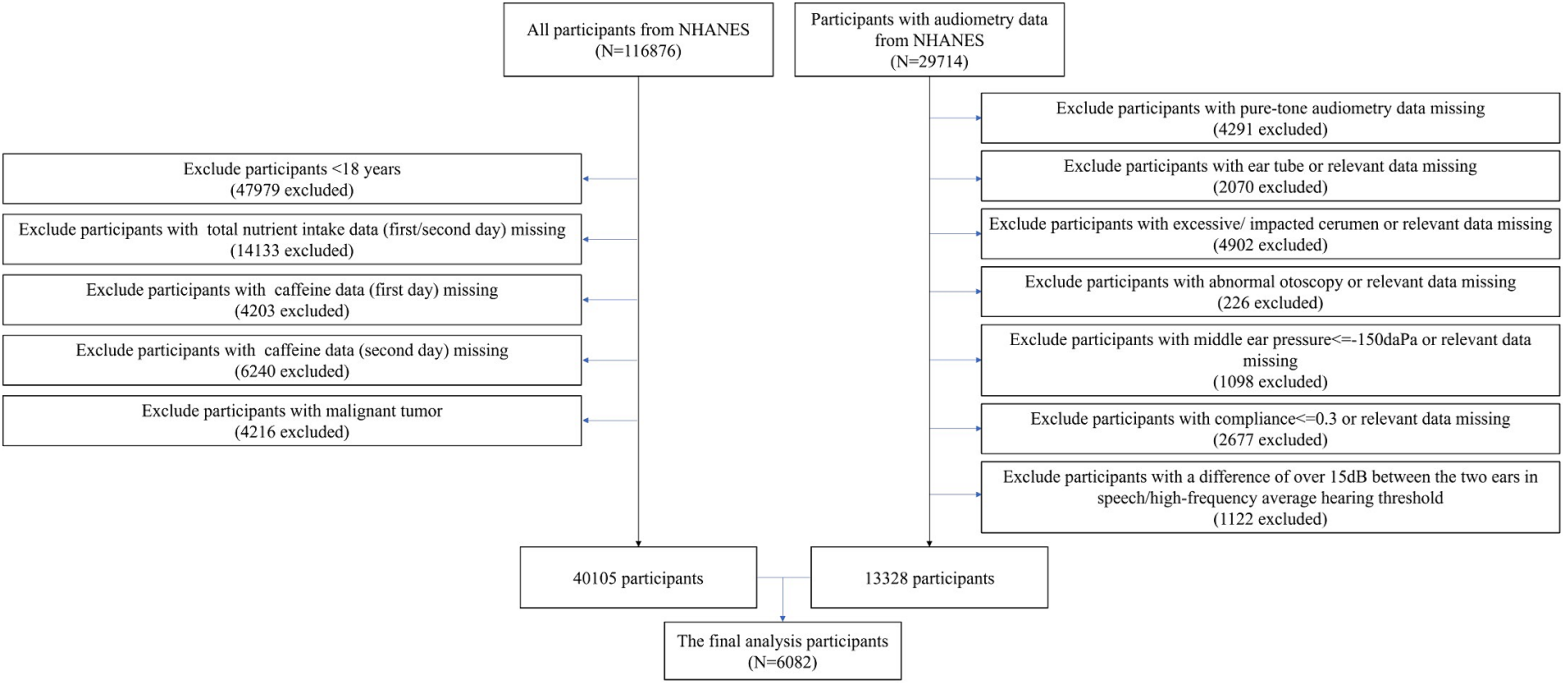
Flow chart of study population.

The NHANES survey, which is a cross-sectional study designed to represent the noninstitutionalized U.S. population, was approved by the National Center for Health Statistics (NCHS) Research Ethics Review Board.^1^ All participants provided informed consent. Data collection in NHANES involves detailed in-person interviews conducted at participants’ homes followed by physical examinations and laboratory tests performed at Mobile Examination Centers (MECs). A total of 116,876 participants from NHANES (1999–2020, pre-pandemic) were initially extracted. Among this cohort, 29,714 participants with available audiometric data were included. The study applied several exclusion criteria: (1) participants under 18 years of age; (2) missing data on total nutrient intake or caffeine consumption for either the first or second day; (3) missing pure-tone audiometry data; (4) participants with excessive or impacted cerumen; (5) abnormal otoscopy findings; (6) middle ear pressure below −150 daPa; (7) compliance below 0.3; (8) a difference of over 15 dB between the ears in speech or high-frequency hearing thresholds; and (9) a history of malignant tumors. Given the complexity and vastness of the public database, we processed the data in segments. Initially, we identified 13,328 participants with complete audiometry data after applying the exclusion criteria. Separately, we processed the dietary data and identified 40,105 participants with complete dietary data who also met the exclusion criteria. After applying the criteria to both datasets, a total of 6,082 participants with complete dietary and audiometry data were included in the study, based on data collected between 2003–2012 and 2015–2020 (pre-pandemic). The exclusion of the period from 2013 to 2014 was due to the unavailability of audiometric data (Figure 1).

### 2.2 Assessment of caffeine intake

All NHANES participants are eligible for two 24-h dietary recall interviews. The first interview is conducted in-person at the Mobile Examination Center (MEC), and the second is conducted by telephone 3 to 10 days later. Data on caffeine intake (mg/day) is obtained from the files “Dietary Interview – Total Nutrient Intakes, First Day” and “Dietary Interview – Total Nutrient Intakes, Second Day,” which compile nutrients from all foods and beverages reported.

Using information from these interviews, we estimated each participant’s average daily caffeine intake from all caffeine-containing foods and beverages, including coffee, tea, soda (regular, reduced calorie, and diet), energy drinks, and chocolate. The caffeine intake data for this study is the average of the values from the two 24-h recall interviews.

This data is entered into the USDA’s Food and Nutrient Database for Dietary Studies (FNDDS), which provides detailed nutrient information, including caffeine content. The caffeine content for each food and beverage is calculated using standardized data from the FNDDS based on reported intake amounts. The total caffeine intake for each participant is then determined by summing the caffeine content from all reported foods and beverages within the 24-h period.

### 2.3 Assessment of HL

The “Audiometry” file from the NHANES examination section provided the audiometric data for this study. Audiometric testing was conducted by qualified examiners in sound-isolating chambers using an AD226 audiometer, TDH-39P headphones, and EARTone 3A earphones.

The hearing test procedures included several steps to ensure accuracy and reliability: a pre-exam audiometric questionnaire to identify conditions affecting testing or results, a brief otoscopic exam to detect ear abnormalities, middle ear testing to measure eardrum compliance and identify pathologies, and pure-tone air conduction audiometry to measure hearing sensitivity.

Quality assurance and control were maintained through various measures: daily and start/end of survey site calibration checks, an annual comprehensive National Institute of Standards and Technology (NIST) traceable calibration check, continuous monitoring of environmental noise levels, automatic data upload and review using a computer program to check data consistency and errors, repeated tests to ensure the reliability of audiograms, and professional training and supervision of health technicians by certified audiologists. National Institute for Occupational Safety & Health (NIOSH) consultants also provided annual retraining and protocol updates to maintain high standards of quality and consistency.

Pure-tone air conduction hearing thresholds were measured at frequencies of 0.5, 1, 2, 3, 4, and 6 kHz, with intensity ranges from −10 to 110 dB. High-frequency hearing loss (HFHL) was evaluated using averages at 3, 4, and 6 kHz, while speech-frequency hearing loss (SFHL) was determined using averages at 0.5, 1, 2, and 4 kHz. HL was defined as a threshold of ≥25 dB in either ear for both SFHL and HFHL (35, 36).

### 2.4 Grouping and outcomes

All participants were divided into low caffeine intake group and high caffeine intake group according to the median caffeine intake level (low caffeine intake: <78 mg/day, *n* = 3,031; high caffeine intake≥78 mg/day, *n* = 3,051). SFHL and HFHL were the main outcomes assessed in this investigation.

### 2.5 Covariates

Information on demographics, education level, ratio of family income to poverty (PIR), smoking status (having smoked at least 100 cigarettes in a lifetime), comorbidities (such as hypertension, diabetes, and hypercholesterolemia), and medication use in the past month (including nonsteroidal anti-inflammatory drugs (NSAIDs), diuretics, and aminoglycosides) was collected through structured questionnaires. Additionally, vital signs, including systolic and diastolic blood pressure, and heart rate, were measured at baseline. Body mass index (BMI) was calculated by dividing weight in kilograms by height in meters squared. The dietary data, encompassing energy, protein, carbohydrate, total sugars, dietary fiber, and total fat, were averaged from two 24-h recall interviews.

### 2.6 Statistical analyses

We used sampling weights, clusters, and strata in our analysis to account for the intricate, multistage probability sampling architecture of the NHANES. Normal-distribution continuous data were displayed as means ± standard deviations (SD), while skewed-distribution continuous variables were displayed as medians with interquartile ranges (IQR). The reporting of categorical variables was done as counts, or percentages. Depending on the distribution of the data, the Independent *T*-test and Mann–Whitney U test were employed to analyze continuous variables, while the χ2 test or Fisher’s exact test was used to compare categorical baseline variables. In order to show the distribution of pure-tone audiometry data (averages of left/right hearing thresholds at speech and high frequencies), we also made histograms and classified them according to the amount of caffeine consumed.

Binary logistic regression was used to evaluate the relationship between the levels of caffeine intake and the outcomes (SFHL and HFHL), with odds ratios (ORs) and 95% confidence intervals (CIs) displayed as the outcomes. The correlation between covariates and HL was confirmed through univariate logistic analysis, and variables with *p* < 0.05 were included in the multivariate logistic regression model. The reference group was the one with low caffeine intake. The methodology required removing one or more variables when there was significant collinearity among them (variance inflation factor [VIF] > 10). However, variables that were essential for improving the model’s relevance or explanatory power were prioritized and retained. Model I was unadjusted. Model II included adjustments for age, sex, race, heart rate, diastolic blood pressure, BMI, education level, and smoking status (at least 100 cigarettes smoked in a lifetime), as well as the use of diuretics, NSAIDs (apart from aspirin), diabetes, protein, carbohydrate, total sugars, and total fat. The variables in the restricted cubic spline (RCS) model matched those in Model II, and it was used to investigate non-linear associations between coffee intake levels and HL. The Akaike Information Criterion (AIC) was used to determine which four knots to use for the RCS model.

The association between caffeine intake levels and HL was examined using subgroup analyses using univariate logistic regression. The *p*-value for the interaction was computed and displayed in a forest plot. R software version 4.2.1 and Stata version 15.0 were used for the statistical analyses. *p*-values below 0.05 were regarded as statistically significant.

## 3 Results

### 3.1 Participant characteristics

The media of caffeine intake level was 78 mg/day. It was found that the high caffeine intake group was older (*p* < 0.001), had a significantly higher percentage of males (*p* < 0.001) and Non-Hispanic Whites (*p* < 0.001), and exhibited higher systolic (*p* < 0.001) and diastolic blood pressure (*p* < 0.001) and higher body mass index (*p* = 0.012). The high caffeine intake group demonstrated a higher level of educational level, with a greater proportion of individuals being college graduates or above (*p* < 0.001). The socioeconomic status, as indicated by a higher PIR, was elevated in the high intake group (*p* < 0.001), as was the prevalence of smoking (*p* < 0.001), hypertension (*p* < 0.001), diabetes (*p* = 0.006), and hypercholesterolemia (*p* < 0.001). Regarding medication use, consumption of NSAIDs (no aspirin) was significantly higher in the high caffeine group (*p* = 0.033), with no significant differences noted in the use of aspirin, diuretics, and aminoglycosides. Moreover, the high caffeine intake group exhibited significantly higher intakes of energy (*p* < 0.001), protein (*p* < 0.001), carbohydrate (*p* < 0.001), total sugars (*p* < 0.001), dietary fiber (*p* = 0.001), and total fat (*p* < 0.001) (Table 1).

**Table 1:** Characteristics of participants stratified by caffeine intake level.

Characteristics	Total (*n* = 6,082)	Caffeine intake level		*p* value
		Low (*n* = 3,031) Caffeine intake <78 mg/day	High (*n* = 3,051) Caffeine intake≥78 mg/day	
**Age (years)**	42.0 ± 18.6	38.3 ± 19.0	45.6 ± 17.5	<0.001
**Gender, *n* (%)**				<0.001
Male	2,752 (45.3)	1,299 (42.9)	1,453 (47.6)	
Female	3,330 (54.8)	1732 (57.1)	1,598 (52.4)	
**Race, *n* (%)**				<0.001
Mexican American	982 (16.2)	565 (18.6)	417 (13.7)	
Other Hispanic	576 (9.5)	279 (9.2)	297 (9.7)	
Non-Hispanic White	2,397 (39.4)	891 (29.4)	1,506 (49.4)	
Non-Hispanic Black	1,437 (23.6)	958 (31.6)	479 (15.7)	
Other Race	690 (11.3)	338 (11.2)	352 (11.5)	
**Vital signs**				
Systolic blood pressure (mmHg)	121.0 ± 16.9	120.1 ± 17.7	122.0 ± 16.9	<0.001
Diastolic blood pressure (mmHg)	69.4 ± 12.1	68.3 ± 12.3	70.5 ± 11.7	<0.001
Heart rate (beats/min)	73.0 ± 11.8	73.3 ± 11.8	72.7 ± 11.7	0.050
**Body mass index (kg/m** ^**2**^ **)**	28.9 ± 7.1	28.7 ± 7.2	29.1 ± 6.8	0.012
**Education level, *n* (%)**				<0.001
Less than high school	1,339 (22.0)	774 (25.5)	565 (18.5)	
High school graduate/GED or equivalent	1,460 (24.0)	782 (25.8)	678 (22.2)	
Some college or AA degree	1,780 (29.3)	827 (27.3)	953 (31.2)	
College graduate or above	1,502 (24.7)	647 (21.4)	855 (28.0)	
**PIR**	2.5 ± 1.6	2.3 ± 1.6	2.7 ± 1.6	<0.001
**Smoked at least 100 cigarettes, *n* (%)**	2,385 (39.2)	898 (29.6)	1,487 (48.7)	<0.001
**Comorbidities, *n* (%)**				
Hypertension	1710 (28.1)	778 (25.7)	932 (30.6)	<0.001
Diabetes	574 (9.4)	255 (8.4)	319 (10.5)	0.006
Hypercholesterolemia	1938 (31.9)	862 (28.4)	1,076 (35.3)	<0.001
**Medication use, *n* (%)**				
NSAIDs				
Aspirin	47 (0.77)	23 (0.79)	23 (0.75)	0.866
Other NSAIDs	335 (5.5)	148 (4.9)	187 (6.1)	0.033
Diuretics	191 (3.1)	97 (3.2)	94 (3.1)	0.790
Aminoglycosides	9 (0.15)	5 (0.16)	4 (0.13)	0.731
Energy (kcal)	2084.2 ± 827.8	2005.9 ± 819.1	2162.0 ± 829.1	<0.001
Protein (gm)	81.4 ± 35.7	79.4 ± 35.9	83.4 ± 35.3	<0.001
Carbohydrate (gm)	253.6 ± 107.2	246.4 ± 105.0	260.8 ± 109.0	<0.001
Total sugars (gm)	111.3 ± 63.7	105.6 ± 58.4	116.9 ± 68.1	<0.001
Dietary fiber (gm)	16.6 ± 9.0	16.3 ± 9.1	17.0 ± 8.9	0.001
Total fat (gm)	79.7 ± 37.8	75.8 ± 36.9	83.5 ± 38.4	<0.001

Continuous variables obeying normal distribution were presented as mean ± SD. Categorical variables were presented as number (percentage). GED, general educational development; PIR, ratio of family income to poverty; NSAIDs, nonsteroidal anti-inflammatory drugs; SD, standard deviation.

### 3.2 Audiometry data of participants

**Figure 2:**
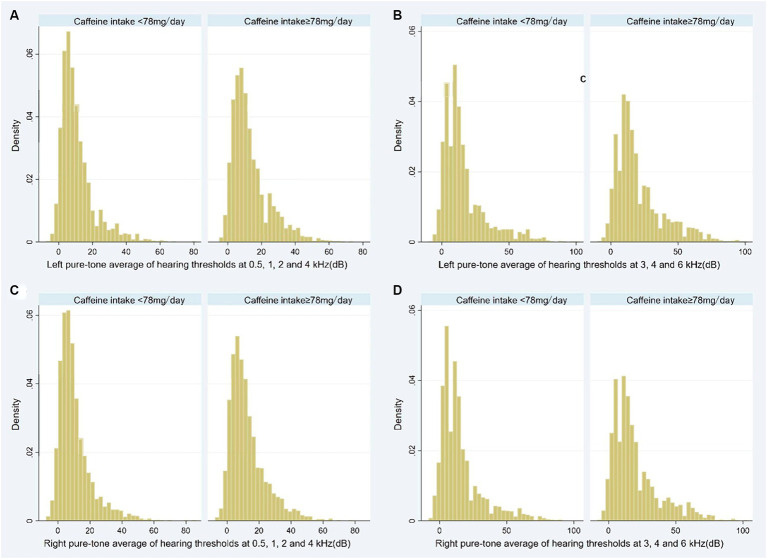
Histogram showing the pure-tone audiometry data of participants stratified by caffeine intake level (**A**: Left pure-tone average of hearing thresholds at 0.5, 1, 2, and 4 kHz; **B**: Left pure-tone average of hearing thresholds at 3, 4, and 6 kHz; **C**: Right pure-tone average of hearing thresholds at 0.5, 1, 2, and 4 kHz; **D**: Right pure-tone average of hearing thresholds at 3, 4, and 6 kHz). dB, decibel.

Participants in high caffeine intake group had higher pure-tone average of hearing thresholds at 0.5, 1, 2 and 4 kHz (Left: low caffeine intake group vs. high caffeine intake group: 8.8 [3.8–15.0] dB vs. 10 [5.0–18.8] dB, *p* < 0.001; Right: low caffeine intake group vs. high caffeine intake group: 7.5 [3.8–13.8] dB vs. 10.0 [5.0–17.5] dB, *p* < 0.001) and higher pure-tone average of hearing thresholds at 3, 4 and 6 kHz (Left: low caffeine intake group vs. high caffeine intake group: 11.7 [5.0–20.0] dB vs. 15.0 [8.3–28.3] dB, *p* < 0.001; Right: low caffeine intake group vs. high caffeine intake group: 10.0 [5.0–20.0] dB vs. 15.0 [8.3–26.7] dB, *p* < 0.001) (Table 2; Figure 2). The incidence of SFHL (low caffeine intake group vs. high caffeine intake group: 302 (10%) vs. 470 (15.4%), *p* < 0.001) and HFHL (low caffeine intake group vs. high caffeine intake group: 624 (20.6%) vs. 930 (30.5%), *p* < 0.001) increased significantly in high caffeine intake group (Table 2).

**Table 2:** Audiometry data of participants stratified by caffeine intake level.

Characteristics	Total (*n* = 6,082)	Caffeine intake level		*p* value
		Low (*n* = 3,031) Caffeine intake <78 mg/day	High (*n* = 3,051) Caffeine intake≥78 mg/day	
**Left (dB)**				
Pure-tone average of hearing thresholds at 0.5, 1, 2, and 4 kHz	8.8 (5.0–16.3)	8.8 (3.8–15.0)	10 (5.0–18.8)	<0.001
Pure-tone average of hearing thresholds at 3, 4, and 6 kHz	13.3 (6.7–23.3)	11.7 (5.0–20.0)	15.0 (8.3–28.3)	<0.001
**Right (dB)**				
Pure-tone average of hearing thresholds at 0.5, 1, 2, and 4 kHz	8.8 (5.0–16.3)	7.5 (3.8–13.8)	10.0 (5.0–17.5)	<0.001
Pure-tone average of hearing thresholds at 3, 4, and 6 kHz	11.7 (6.7–23.3)	10.0 (5.0–20.0)	15.0 (8.3–26.7)	<0.001
**HL, *n* (%)**				
SFHL	772 (12.7)	302 (10.0)	470 (15.4)	<0.001
HFHL	1,554 (25.6)	624 (20.6)	930 (30.5)	<0.001

Variables in skewness distribution were presented as median (interquartile range). Categorical variables were presented as number (percentage). dB, decibel; HL, hearing loss; SFHL, speech-frequency hearing loss; HFHL, high-frequency hearing loss.

### 3.3 Logistic analysis exploring the association between HL and caffeine intake level

In logistic Model I, no variables were adjusted, the risk of SFHL (OR, 95% CIs: 1.65, 1.41–1.92, *p* < 0.001) and HFHL (OR, 95% CIs: 1.69, 1.50–1.90, *p* < 0.001) significantly increased in high caffeine intake group. In multivariable logistic Model II, the risk of SFHL (OR, 95% CIs: 1.35, 1.09–1.66, *p* = 0.005) still significantly increased in high caffeine intake group. However, the association between caffeine intake and HFHL did not show a statistically significant difference (OR, 95% CIs: 1.14, 0.96–1.35, *p* = 0.144) (Table 3).

**Table 3:** Logistic analysis exploring the association between HL and caffeine intake level.

	Model I		Model II	
	OR (95% CIs)	*p* value	OR (95% CIs)	*p* value
**SFHL**				
Caffeine intake <78 mg/day	1.0 (Ref)		1.0 (Ref)	
Caffeine intake≥78 mg/day	1.65 (1.41–1.92)	<0.001	1.35 (1.09–1.66)	0.005
**HFHL**				
Caffeine intake <78 mg/day	1.0 (Ref)		1.0 (Ref)	
Caffeine intake≥78 mg/day	1.69 (1.50–1.90)	<0.001	1.14 (0.96–1.35)	0.144

Models were derived from logistic regression analysis. Model I: unadjusted. Model II: adjusted for age, sex, race, diastolic blood pressure, heartrate, body mass index, education level, smoked at least 100 cigarettes, diabetes, diuretics, NSAIDs (no aspirin), protein, carbohydrate, total sugars, and total fat. HL, hearing loss; OR, odds ratio; CI, confidence interval; SFHL, speech-frequency hearing loss; HFHL, high-frequency hearing loss; NSAIDs, nonsteroidal anti-inflammatory drugs.

### 3.4 RCS model showing the association between the caffeine intake level and HL

**Figure 3:**
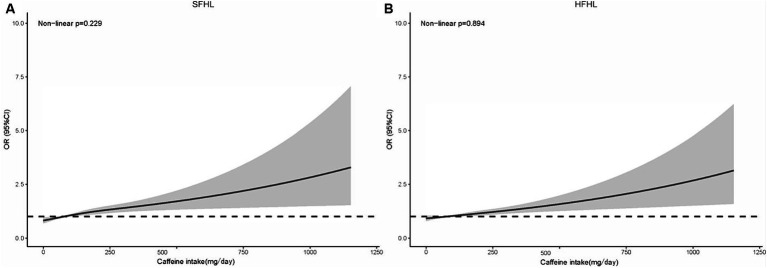
RCS model showing the association between the caffeine intake level and HL (**A**: SFHL; **B**: HFHL). RCS, restricted cubic spline; HL, hearing loss; OR, odds ratio; CI, confidence interval; SFHL, speech-frequency hearing loss; HFHL, high-frequency hearing loss.

The RCS model in line with model II showed that the risk of SFHL (non-linear *p* = 0.229) and HFHL (non-linear *p* = 0.894) increased as the caffeine intake value increased (Figure 3).

### 3.5 Forest plot showing the association between the caffeine intake level and HL in different subgroups

**Figure 4:**
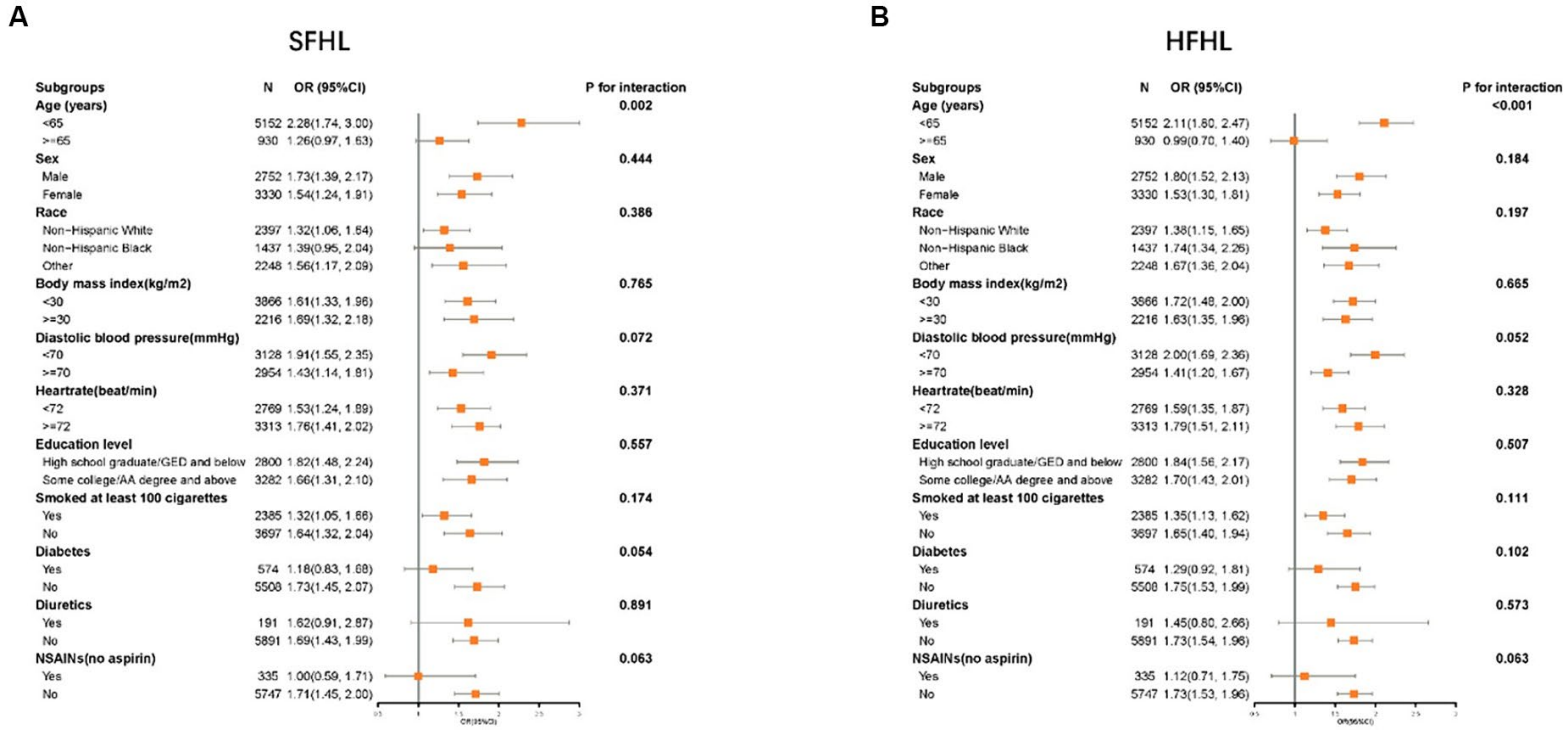
Forest plot showing the association between the caffeine intake level and HL in different subgroups (**A**: SFHL; **B**: HFHL). SFHL: speech-frequency hearing loss; HFHL: high-frequency hearing loss; OR: odds ratio; CI: confidence interval; NSAIDs: nonsteroidal anti-inflammatory drugs; HL: hearing loss.

Subgroup analysis confirmed the higher risk of SFHL (*p* for interaction = 0.002) and HFHL (*p* for interaction<0.001) with caffeine intake in a subgroup of age < 65, there is no obvious statistical significance in subgroup of age ≥ 65. No obvious interaction was observed in other subgroups (Figure 4).

## 4 Discussion

This study focused on American adults from the NHANES dataset, exploring the association between caffeine intake levels and HL. The major findings were as follows: (1) High caffeine intake was significantly associated with the elevated risk of SFHL even after adjusting for possible confounding risk factors, yet showed no significant link with HFHL; (2) The RCS model showed that the risk of SFH and HFHL increased as the caffeine intake value increased and there was a linear relationship (non-linear *p* > 0.05); and (3) Significant interaction effect was observed in subgroup of age.

HL, the third leading cause of disability, affected 157 million individuals globally in 2019, and projections indicated that by 2050, 2.45 billion people will experience HL (1, 37). The large and rapidly growing population necessitates our attention to the issue of HL. It is crucial to identify relevant risk factors and implement corresponding preventive measures. However, traditional risk factors such as aging, genetic mutations, noise exposure, and ototoxic side effects from certain medications only account for a portion of the causes of HL (4, 12). Recently, there has been increasing focus on non-traditional risk factors, including lifestyle and dietary habits (14, 38).

Caffeine, widely recognized for its stimulating effects on the central nervous system (CNS), is one of the most consumed psychoactive substances globally (29, 39). And previous studies indicated that it played a role in the peripheral and central auditory system, while also directly affecting the inner ear (31–34). Caffeine has been shown to impact hearing negatively through its antagonistic effects on adenosine receptors, particularly following acoustic trauma (40). It impedes cochlear blood reperfusion, increases oxidative stress, exacerbates cochlear hair cells’ apoptosis via calcium accumulation (40–43), and heightens noise-induced cochlear hypoperfusion and ischemia by promoting the reduction of cerebral blood flow and arteriole diameter (44, 45). Moreover, caffeine elicits an acute auditory response to noise by affecting corticosterone levels and triggers autophagy and apoptosis in cochlear hair cells through SGK1/HIF-1α pathway (46–48). These actions collectively suggest caffeine’s potential to interfere with hearing recovery processes and contribute to auditory system disorders. The aforementioned mechanisms may explain the correlation between dietary intake of caffeine and HL.

Currently, no studies have specifically identified a relationship between dietary intake of caffeine and hearing loss (HL). Previous research has explored urinary caffeine metabolites and found no association with hearing threshold shifts in US adults (49). Several studies have investigated the relationship between coffee consumption and HL, but they have yet to reach a consensus (35, 50–52). A cross-sectional analysis including 1894 adult individuals showed that coffee consumption was related to higher prevalence of HL (35). In a population-based cohort study involving 36,923 participants, it was discovered that coffee consumption correlated with a reduced risk of developing disabling hearing impairment among men, while no significant association was observed for women. This discrepancy was attributed to differences between men and women in brain biochemistry, cochlear structure, the progression of age-related hearing loss, estrogen levels, and antioxidant responses. Additionally, obese men, who had higher levels of inflammation and oxidative stress, experienced more pronounced protective effects from coffee (50). Similarly, a study based on a national population-based survey revealed that regular coffee drinkers experienced a 50–70% reduction in hearing loss compared to infrequent consumers, suggesting a relationship that appears to be dependent on dosage (51). The variation in research outcomes may be attributed to differences in the ethnic backgrounds of the study populations (Americans, British, Koreans). Additionally, it was possible that the complex composition of coffee, which includes proteins, fats, tannins, caffeine, minerals, and other trace ingredients, contributes to varying outcomes, as the proportional composition of these components differs across regions (52).

Therefore, our study, focused on the American population and analyzing solely the caffeine content, concluded that high dietary intake of caffeine was associated with an increased risk of HL. However, as this investigation is a cross-sectional study, it cannot establish causality between these variables. Large-scale, prospective studies are required to confirm this conclusion. Furthermore, our study revealed that in the multivariate logistic regression, there was no statistically significant difference in the correlation between dietary caffeine intake and HFHL. However, upon reanalysis, when covariates other than age were included, a significant correlation between dietary caffeine intake and HFHL was observed (OR, 95% CIs: 1.61, 1.42–1.83, *p* < 0.001). Age-related HL develops from the aging process’s cumulative effects on the auditory system, characterized by a gradual, bilateral, and symmetrical decrease in hearing sensitivity, especially at higher frequencies (53). Age-related HL affects high frequencies initially and then progresses to lower frequencies over time (54). This pattern suggests that HFHL is often the first to be affected as individuals age. These could explain why, in a multiple regression analysis, after adding age as a covariate, the correlation between caffeine intake and SFHL remained statistically significant, while the correlation with HFHL no longer did. In subgroup analysis, we uncovered an interesting phenomenon: there was a significant correlation between caffeine intake and HL in the subgroup under 65 years of age, but no significant correlation was found in the subgroup aged 65 and older. This indicated that within the older population, HL was predominantly influenced by age, with the contribution of dietary factors becoming less significant. In our research, the prevalence of SFHL and HFHL among individuals under the age of 65 was documented at 4.8 and 15.1%, respectively. Such elevated incidence rates highlighted the critical need for intervention. Consequently, for individuals younger than 65, particularly those exposed to risk factors for HL like noise, modifying dietary habits to decrease caffeine consumption was considered crucial.

The data for our study were obtained from the NHANES, a long-standing program in the USA characterized by its rigorous and mature survey methodology. This foundation enhanced the reliability of our findings. Moreover, the intricate design of NHANES, which includes complex, multistage, probability sampling, necessitated the use of sampling weights in our statistical analyses. Such measures were crucial in mitigating biases associated with such data, further bolstering the credibility of our results. This study also presented a number of limitations: (1) Due to the inherent nature of an observational cross-sectional study, this research is only capable of exploring correlations and cannot establish definitive causality. This constitutes one of the study’s limitations, necessitating large-scale prospective studies to confirm the reliability of the findings. (2) The acquisition of data on caffeine intake was performed via questionnaire surveys, potentially leading to recall bias among participants when documenting their consumption of caffeine-containing foods and beverages. This presents a challenge in accurately capturing detailed information on caffeine intake levels. Therefore, the discrepancy between the assessed exposure and the true exposure to caffeine intake cannot be entirely eradicated. (3) Differences in metabolism between individuals can also potentially impact the relationship between caffeine intake and HL.

## 5 Conclusion

The risk of SFHL and HFHL significantly increased in high caffeine intake group. But after adjusting for confounding variables, high caffeine intake was still associated with the elevated risk of SFHL, yet showed no significant link with HFHL. The RCS model showed that the risk of SFHL and HFHL increased positively and linearly with caffeine intake. A significant interaction was observed in the age subgroup. The results of this study underscore the importance of dietary caffeine intake in the management of HL, particularly in individuals younger than 65 years. However, large-scale prospective studies are needed to confirm the reliability of these findings.

## Data availability statement

Publicly available datasets were analyzed in this study. This data can be found here: the National Health and Nutrition Examination Survey (NHANES) database (https://www.cdc.gov/nchs/nhanes/index.htm).

## Ethics statement

The studies involving humans were approved by the Ethics Committee of Beijing Chaoyang Hospital. The studies were conducted in accordance with the local legislation and institutional requirements. Written informed consent for participation was not required from the participants or the participants’ legal guardians/next of kin in accordance with the national legislation and institutional requirements.

## Author contributions

FX: Writing – original draft, Methodology. YR: Writing – review & editing, Formal analysis.

## Footnotes

^1^ http://www.cdc.gov/nchs/nhanes
